# Supplementary material for: The Inhibitory Receptor GPR56 (Adgrg1) Is Specifically Expressed by Tissue-Resident Memory T Cells in Mice But Dispensable for Their Differentiation and Function In Vivo
Source: Cells. 2021 Oct 6;10(10):2675. doi: 10.3390/cells10102675 (PMC8534179; doi:10.3390/cells10102675)
Supplement: Supplementary file 1 [file cells-10-02675-s001.zip › cells-1405532.pptx]

## Slide 1
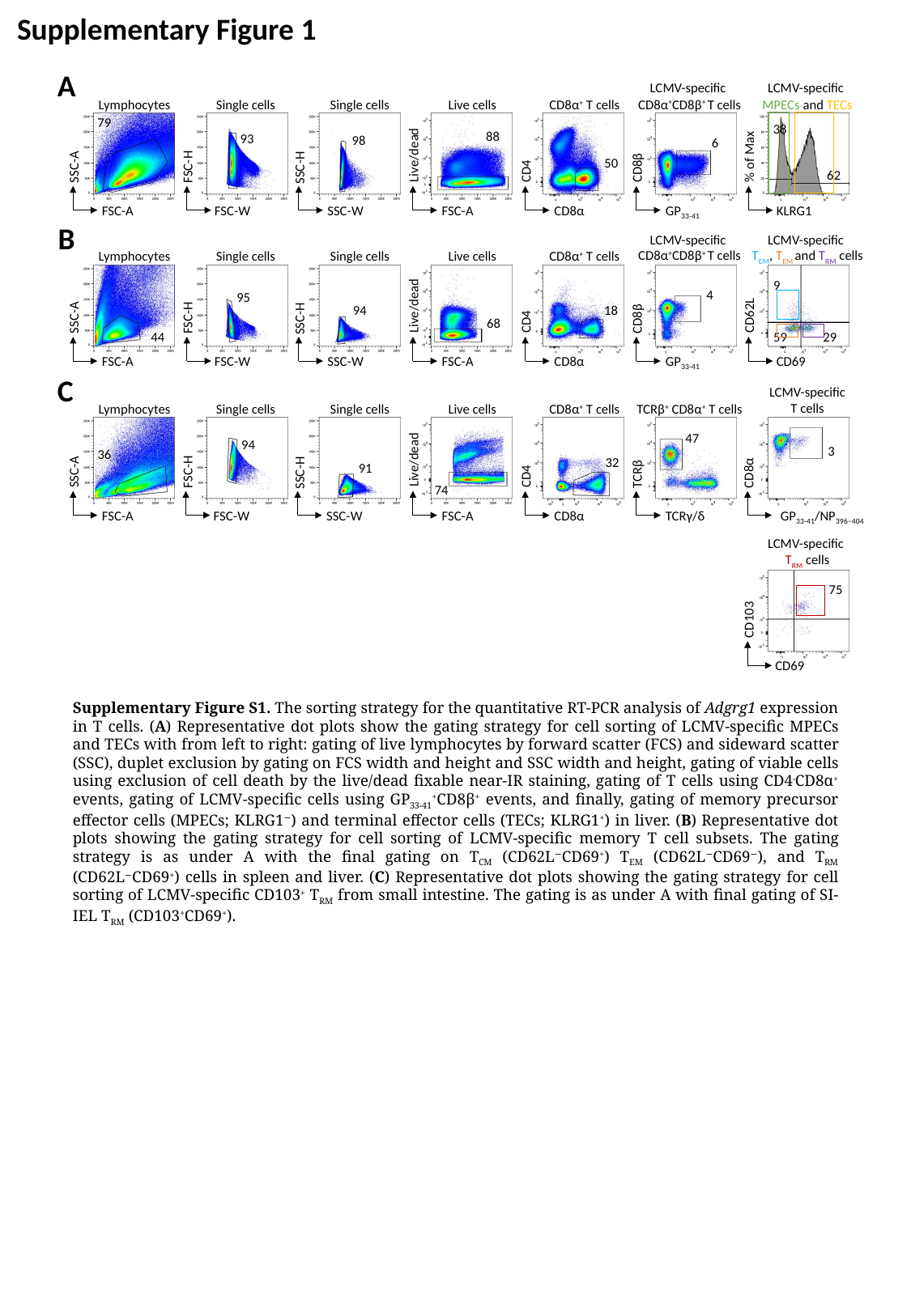

Supplementary Figure 1
A
LCMV-specific
CD8α+CD8β+ T cells
LCMV-specific
MPECs and TECs
Lymphocytes
Single cells
Single cells
Live cells
CD8α+ T cells
79
38
Live/dead
FSC-A
% of Max
KLRG1
88
93
98
6
FSC-H
FSC-W
SSC-A
FSC-A
SSC-H
SSC-W
CD8β
GP33-41
CD4
CD8α
50
62
B
LCMV-specific
CD8α+CD8β+ T cells
LCMV-specific
TCM, TEM and TRM cells
Lymphocytes
Single cells
Single cells
Live cells
CD8α+ T cells
Live/dead
FSC-A
9
4
95
CD62L
CD69
FSC-H
FSC-W
SSC-A
FSC-A
SSC-H
SSC-W
CD8β
GP33-41
18
94
CD4
CD8α
68
59
29
44
C
LCMV-specific T cells
Lymphocytes
Single cells
Single cells
Live cells
CD8α+ T cells
TCRβ+ CD8α+ T cells
Live/dead
FSC-A
47
94
3
36
FSC-H
FSC-W
SSC-A
FSC-A
SSC-H
SSC-W
CD8α
GP33-41/NP396−404
TCRβ
TCRγ/δ
32
CD4
CD8α
91
74
LCMV-specific
TRM cells
75
CD103
CD69
Supplementary Figure S1. The sorting strategy for the quantitative RT-PCR analysis of Adgrg1 expression in T cells. (A) Representative dot plots show the gating strategy for cell sorting of LCMV-specific MPECs and TECs with from left to right: gating of live lymphocytes by forward scatter (FCS) and sideward scatter (SSC), duplet exclusion by gating on FCS width and height and SSC width and height, gating of viable cells using exclusion of cell death by the live/dead fixable near-IR staining, gating of T cells using CD4-CD8α+ events, gating of LCMV-specific cells using GP33-41+CD8β+ events, and finally, gating of memory precursor effector cells (MPECs; KLRG1−) and terminal effector cells (TECs; KLRG1+) in liver. (B) Representative dot plots showing the gating strategy for cell sorting of LCMV-specific memory T cell subsets. The gating strategy is as under A with the final gating on TCM (CD62L−CD69+) TEM (CD62L−CD69−), and TRM (CD62L−CD69+) cells in spleen and liver. (C) Representative dot plots showing the gating strategy for cell sorting of LCMV-specific CD103+ TRM from small intestine. The gating is as under A with final gating of SI-IEL TRM (CD103+CD69+).

## Slide 2
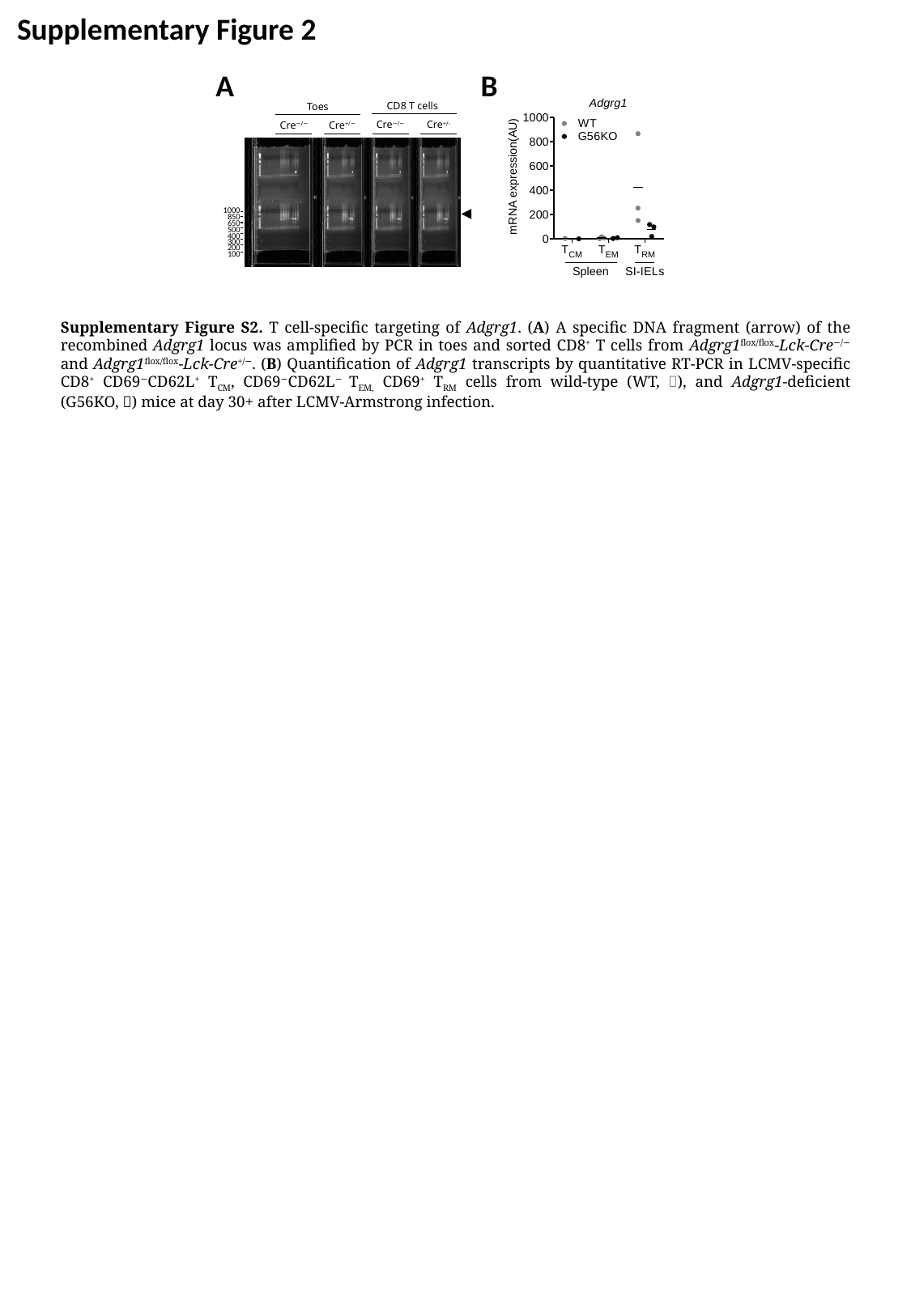

Supplementary Figure 2
A
B
CD8 T cells
Toes
Cre−/−
Cre+/-
Cre−/−
Cre+/−
1000
850
650
500
400
300
200
100
Supplementary Figure S2. T cell-specific targeting of Adgrg1. (A) A specific DNA fragment (arrow) of the recombined Adgrg1 locus was amplified by PCR in toes and sorted CD8+ T cells from Adgrg1flox/flox-Lck-Cre−/− and Adgrg1flox/flox-Lck-Cre+/−. (B) Quantification of Adgrg1 transcripts by quantitative RT-PCR in LCMV-specific CD8+ CD69−CD62L+ TCM, CD69−CD62L− TEM, CD69+ TRM cells from wild-type (WT, =), and Adgrg1-deficient (G56KO, =) mice at day 30+ after LCMV-Armstrong infection.
